# Comparisons of individual- and area-level socioeconomic status as proxies for individual-level measures: evidence from the Mortality Disparities in American Communities study

**DOI:** 10.1186/s12963-020-00244-x

**Published:** 2021-01-07

**Authors:** Jennifer L. Moss, Norman J. Johnson, Mandi Yu, Sean F. Altekruse, Kathleen A. Cronin

**Affiliations:** 1 Cancer Prevention Fellowship Program, Surveillance Research Program, Division of Cancer Control and Population Sciences, National Cancer Institute, Bethesda, MD USA; 2 Department of Family and Community Medicine, Penn State College of Medicine, The Pennsylvania State University, 134 Sipe Ave., #205, P.O. Box 850, MC HS72, Hershey, PA 17033 USA; 3 Center for Administrative Records Research and Applications, Research & Methodologies Directorate, US Bureau of the Census, Suitland, MD USA; 4 Statistical Research & Applications Branch, Surveillance Research Program, Division of Cancer Control and Population Sciences, National Cancer Institute, Bethesda, MD USA; 5 Cardiovascular Epidemiology Branch, National Heart, Lung, and Blood Institute, Bethesda, MD USA; 6 Office of the Associate Director, Surveillance Research Program, Division of Cancer Control and Population Sciences, National Cancer Institute, Bethesda, MD USA

**Keywords:** Socioeconomic status, Individuals, Census tracts, Counties, Mortality, Social epidemiology

## Abstract

**Background:**

Area-level measures are often used to approximate socioeconomic status (SES) when individual-level data are not available. However, no national studies have examined the validity of these measures in approximating individual-level SES.

**Methods:**

Data came from ~ 3,471,000 participants in the Mortality Disparities in American Communities study, which links data from 2008 American Community Survey to National Death Index (through 2015). We calculated correlations, specificity, sensitivity, and odds ratios to summarize the concordance between individual-, census tract-, and county-level SES indicators (e.g., household income, college degree, unemployment). We estimated the association between each SES measure and mortality to illustrate the implications of misclassification for estimates of the SES-mortality association.

**Results:**

Participants with high individual-level SES were more likely than other participants to live in high-SES areas. For example, individuals with high household incomes were more likely to live in census tracts (*r* = 0.232; odds ratio [OR] = 2.284) or counties (*r* = 0.157; OR = 1.325) whose median household income was above the US median. Across indicators, mortality was higher among low-SES groups (all *p* < .0001). Compared to county-level, census tract-level measures more closely approximated individual-level associations with mortality.

**Conclusions:**

Moderate agreement emerged among binary indicators of SES across individual, census tract, and county levels, with increased precision for census tract compared to county measures when approximating individual-level values. When area level measures were used as proxies for individual SES, the SES-mortality associations were systematically underestimated. Studies using area-level SES proxies should use caution when selecting, analyzing, and interpreting associations with health outcomes.

## Background

Socioeconomic status (SES) is an individual’s relative position within a social hierarchy reflecting their ability to consume resources [1]. In public health research studies, SES is often measured using income, education, and/or occupation [1, 2], but little consensus exists in the proper definition and measurement of SES [3]. For decades, research has linked SES to health, generally concluding that higher SES is associated with better health [4–8].

However, SES data are sensitive. In many population-based datasets, individual-level SES is not available. Furthermore, when studies request this information, participants may decline to respond or exaggerate their SES [9, 10]. To address this challenge, researchers often use area-level measures to approximate individual-level SES. For example, instead of measuring an individual’s income, education, or occupation, a researcher can link that individual to a geographically defined area (e.g., their census tract or county) and measure the median household income, percent of residents with at least a high school degree, or percent of residents in blue-collar occupations [3, 11]. These measures of area-level SES are publicly available from nationally representative federal resources (e.g., the American Community Survey (ACS) of the US Census Bureau [12]). As with individual-level studies, research that uses aggregated measures generally find that higher area-level SES is associated with better health [13–16].

Area-level SES measures are used in at least two ways in public health research: (1) as proxies for individual-level SES [17–19] (as described above) and (2) as indicators of the environment, which affects health independent of individual SES [20–22]. Previously, studies have found fair agreement between SES characteristics across socioecological levels [23–29]; however, most of these studies have taken place in the context of relatively small geographic areas. Further, the extent of misclassification of individual- versus area-level SES when estimating associations between SES and health is unclear.

To evaluate the validity of area-level characteristics as proxies for individual-level SES, we analyzed concordance between individual-, census tract-, and county-level SES in the nationwide Mortality Disparities in American Communities (MDAC) study, which includes data for more than 4.5 million people in the US. We sought to illustrate the impact of misclassification when area-level characteristics are used to estimate health disparities by SES.

## Methods

### Data source

Data were obtained from MDAC, a project by the US Census Bureau, Centers for Disease Control, and National Institutes of Health to facilitate research on mortality disparities by social and economic characteristics (https://www.census.gov/mdac.html). The construction of MDAC involved linking the data from the 2008 ACS to mortality data obtained through the use of the National Death Index (NDI) and other sources for 2008 to 2015. Only ACS records which have the necessary information available to match to NDI records are maintained in the MDAC study’s official research database. The sampling frame for ACS is derived from the Census Bureau’s Master Address File, a continuously updated file of the addresses of known living quarters (both regular housing units and group quarters) in the US. The sampling scheme for the 2008 ACS is a complex stratified sample of the US population conducted on a yearly basis. When 5 years of ACS data are combined, they allow robust SES attribute estimates at the block, census tract, and county level. These estimates have replaced population estimates that previously came from the Decennial Census long form. Further details about the design of ACS are available in Chapter 4 of the ACS Design and Methodology document (https://www.census.gov/programs-surveys/acs/methodology/design-and-methodology.html).

For MDAC purposes, ACS weights were reweighted by age, sex, race, Hispanic status, and state to the US population to account for the ACS records in MDAC which were not successfully linked to NDI. For the purposes of this paper, analyses were restricted to persons of age 18 years or older (*n* ≈ 3,471,000) linked to a census tract (*n* ≈ 2,830,000) or county (*n* ≈ 2,854,000). The individual-level MDAC records were linked to 5-year estimates of area-level characteristics from ACS (2006–2010, i.e., centered around 2008). MDAC data came from participants in 3242 US counties and 73,057 census tracts in the USA.

The Office of Management and Budget approved data collection for ACS. The procedures for the current analysis were approved by the Center for Economic Studies at the US Census Bureau. Output was reviewed by the US Census Bureau staff to maintain confidentiality of human subjects’ data.

### Measures

#### Socioeconomic status

We gathered widely used characteristics of SES [1, 30, 31] for individuals, census tracts, and counties. Several of the individual-level characteristics (e.g., employment status) are necessarily binary. To increase comparability across levels and categories, we dichotomized all continuous SES characteristics at the US median for each socioecological level (i.e., *less than or equal to* the median versus *above* the median for individuals, census tracts, or counties). We used these binary variables of individual- and area-level SES characteristic to examine high- versus low-risk groups for adverse health outcomes.

##### Household income

We dichotomized each participant’s individual-level household income compared to the US median household income. In addition, we dichotomized area-level (census tract and county) indicators of median household income compared to US median.

##### Poverty

We developed individual-level household poverty indicators based on whether each participant’s household was below 100% of the federal poverty level (calculated based on household income and family size [32]). In addition, we dichotomized area-level indicators of percentage of households below the federal poverty level compared to US median.

##### Education

We developed individual-level educational indicators based on whether each participant (a) had a high school degree, restricted to participants who were 18+ years old, and (b) had a 4-year college degree, restricted to participants 25+ years old. In addition, we dichotomized area-level indicators of percentage of relevant populations that had reached each educational milestone compared to US median.

##### Employment/occupation

We developed individual-level employment/occupational indicators based on whether each participant was (a) unemployed versus employed, restricted to participants who were in the workforce, and (b) employed in a blue-collar industry (including transportation, repair, and service industries) [33] versus other occupations, restricted to participants who were employed. In addition, we dichotomized area-level indicators of the percentage of relevant populations in each employment/occupational category compared to the US median.

##### Other

We developed individual-level indicators of other, less frequently used markers of SES: whether participants owned the home they lived in and whether they were born in a different country. In addition, we dichotomized area-level indicators of the percentage of the population in each category compared to the US median.

#### Mortality

Individual-level mortality data indicated whether each MDAC participant had died from any cause over the 7-year follow-up (through December 31, 2015).

### Statistical analysis

We calculated each SES characteristic’s proportion and standard deviation to describe the distribution across socioecological levels (note that, for area-level proportions which are definitionally ~ 0.50, standard deviation, calculated as $\sqrt{p\ast \left(1-p\right)}$, must also be ~ 0.50). Then, we estimated the correlations among individual-, census tract-, and county-level SES characteristics using Spearman’s correlation coefficients. Correlations between individual- and area-level estimates are necessarily attenuated because extreme individual-level observations are smoothed in summary measures. Imperfect correlations are expected because area-level proportions are rarely 0% or 100%, while individual-level indicators are always either 0 or 1. Correlations would only reach the theoretically possible bounds of − 1 or + 1 if all individuals within an area had the same value. For example, even in counties that are above the median for unemployment (i.e., in the high-risk county-level SES category as measured by unemployment), most individuals within these counties would still be employed. Although some researchers [34, 35] have pointed out this limitation, the empirical implications have not been described.

Next, we generated cross classifications of each characteristic and calculated sensitivity and specificity comparing area-level SES characteristics to the “gold standard” of individual-level SES [22, 26]. Then, we used generalized estimating equations to examine the associations between individual- and area-level SES characteristics. We created models predicting each individual-level SES attribute using the census tract- and county-level SES attributes simultaneously, adjusting for the clustering of individuals within census tracts and counties.

Finally, to demonstrate the relevance of misclassification of SES across socioecological levels for public health research, we calculated estimates of the SES-mortality association. We constructed logistic models examining the association of each individual-, census tract-, and county-level SES measure (separately) with mortality.

Analyses were conducted in SAS version 9.3 (Cary, NC). Individual-level observations were weighted to account for non-equal probability of selection into ACS/MDAC and to increase generalizability. Below, we present unweighted frequencies and weighted proportions. Models included a control variable for county population (measured in 1000s of population). Because of the exceptionally large sample size, we generally do not present confidence intervals or *p* values in the tables.

## Results

The proportion and standard deviation (*SD*) of SES characteristics for individuals, census tracts, and counties appear in Table 1.

**Table 1:** Proportions of individuals in each socioeconomic status (SES) category and percent of tracts and counties in the category relative to the US median, Mortality Disparities in American Communities study

	Individual-level (n ≈ 3,471,000)		Census tract-level (***n*** = 2,830,000)		County-level (***n*** = 2,854,000)	
	Proportion	SD	Proportion compared to the US median	SD	Proportion compared to the US median	SD
Household income						
Above US median	0.47	0.50	0.51	0.50	0.49	0.50
Poverty level						
≤ 100% FPL	0.10	0.30	0.49	0.50	0.46	0.50
Education						
Has high school degree^a^	0.86	0.35	0.50	0.50	0.52	0.50
Has college degree^b^	0.26	0.44	0.47	0.50	0.48	0.50
Employment/occupation						
Is unemployed^c^	0.04	0.18	0.49	0.50	0.48	0.50
Employed in blue-collar industry^d^	0.37	0.48	0.52	0.50	0.53	0.50
Other characteristics						
Owns home	0.74	0.44	0.55	0.50	0.53	0.50
Foreign born	0.13	0.33	0.46	0.50	0.46	0.50

Individual-level SES categories are binary variables based on individual-level values, while census tract- and county-level SES categories reflect whether the areas were at or below versus above the US median for that characteristic. For household income, education, and owns home, having a value of 1 was considered low risk for health outcomes, while having a value of 0 was considered high risk; in contrast, for poverty, employment/occupation, and foreign born, having a value of 1 was considered high risk, while having a value of 0 was considered low risk

*SD* standard deviation, *US* United States, *FPL* federal poverty level

^a^Having a high school degree was measured among participants who were 18+ years old

^b^Having a college degree was measured among participants who were 25+ years old

^c^Unemployment was measured among participants who reported being in the workforce (e.g., excluding retirees)

^d^Employment in a blue-collar industry was measured among participants who reported being employed (i.e., excluding unemployed participants)

### Correlations and concordance of individual- and area-level SES characteristics

Correlations between individual- and area-level SES characteristics were small (Table 2; all *p* < .0001). For example, having an individual-level household income above the median for US households was correlated with living in (1) a census tract that had a median household income above the median for the US census tracts (Spearman’s *r* = .232) and (2) a county that had a median household income above the median for the US counties (Spearman’s *r* = .157). The correlations between individual- and census tract-level characteristics ranged from 0.048 (for unemployment) to 0.263 (for foreign born), and the correlations between individual- and county-level characteristics ranged from 0.028 (for unemployment) to 0.245 (for foreign born).

**Table 2:** Spearman’s correlation coefficients for associations between individual-level SES characteristics and census tract- and county-level SES characteristics, Mortality Disparities in American Communities study

Individual-level characteristic	Census tract-level characteristic	County-level characteristic
	*r*	*r*
Household income		
Above US median	0.232	0.157
Poverty level		
≤ 100% FPL	0.122	0.067
Education		
Has high school degree	0.158	0.096
Has college degree	0.225	0.150
Employment/occupation		
Is unemployed^a^	0.048	0.028
Employed in blue-collar industry^b^	0.070	0.032
Other characteristics		
Owns home	0.230	0.119
Foreign born	0.263	0.245

For household income, education, and owns home, having a value of 1 was considered low risk for health outcomes, while having a value of 0 was considered high risk; in contrast, for poverty, employment/occupation, and foreign born, having a value of 1 was considered high risk, while having a value of 0 was considered low risk. All *p* < .0001

*US* United States, *FPL* federal poverty level

^a^Unemployment was measured among participants who reported being in the workforce (e.g., excluding retirees)

^b^Employment in a blue-collar industry was measured among participants who reported being employed (i.e., excluding unemployed participants)

The cross-classifications of SES categories for individual- with census tract- and county-level characteristics appear in Table 3. For example, 32% of participants lived in households as well as census tracts with incomes less than/equal to the US median (specificity = 60%), while 30% lived in households as well as census tracts above the median (sensitivity = 63%). The remaining 38% of participants had a household income that was misclassified by their census tract’s characteristic. Specifically, 21% had low household incomes but lived in census tracts above the US median, and 17% had high household incomes but lived in census tracts less than/equal to the US median. Similarly, 31% of participants lived in both households and counties with incomes less than/equal to the median (specificity = 58%), while 27% lived in both households and counties above the median (sensitivity = 57%). Across characteristics, specificity for census tracts was 51–69% and for counties was 48–60%. Sensitivity for census tracts was 53–80% and for counties was 54–78%.

**Table 3:** Measures of agreement between individual-level SES characteristics and census tract- and county-level SES characteristics, Mortality Disparities in American Communities study

	Census tract-level characteristic				County-level characteristic			
	Less than/equal to median	Above median	Spec.	Sens.	Less than/equal to median	Above median	Spec.	Sens.
**Individual-level characteristic**								
Household income								
Less than/equal to US median	32%	21%	60%	63%	31%	22%	58%	57%
Above US median	17%	30%			20%	27%		
≤ 100% FPL								
No	48%	42%	53%	67%	50%	40%	55%	56%
Yes	3%	7%			4%	6%		
Has high school degree								
No	10%	4%	69%	53%	9%	6%	60%	54%
Yes	40%	46%			40%	46%		
Has college degree								
No	44%	30%	59%	66%	42%	32%	57%	61%
Yes	9%	17%			10%	16%		
Is unemployed^a^								
No	50%	47%	52%	61%	51%	46%	53%	55%
Yes	1%	2%			2%	2%		
Employed in blue-collar industry^b^								
No	32%	31%	51%	56%	30%	33%	48%	55%
Yes	16%	21%			17%	20%		
Owns home								
No	17%	10%	64%	61%	15%	12%	56%	57%
Yes	28%	45%			31%	42%		
Foreign born								
No	52%	36%	59%	80%	51%	36%	59%	78%
Yes	3%	10%			3%	10%		

For household income, education, and owns home, having a value of 1 was considered low risk for health outcomes, while having a value of 0 was considered high risk; in contrast, for poverty, employment/occupation, and foreign born, having a value of 1 was considered high risk, while having a value of 0 was considered low risk

*Spec.* specificity, *Sens.* sensitivity, *FPL* federal poverty level

^a^Unemployment was measured among participants who reported being in the workforce (e.g., excluding retirees)

^b^Employment in a blue-collar industry was measured among participants who reported being employed (i.e., excluding unemployed participants)

### Relationships among individual- and area-level SES characteristics

Individual-level SES characteristics were closely linked to census tract- and county-level characteristics (Table 4; all *p* < .0001). For example, participants were more likely to have a household income above the US median if they also lived in a census tract (odds ratio [OR] = 2.284) or county (OR = 1.325) whose median household income was above the US median, compared to participants living in low-income census tracts or counties. For all individual-level SES characteristics, the relationship with the corresponding census tract-level characteristic was stronger than that observed for the corresponding county-level characteristic.

**Table 4:** Associations between individual- and census tract- and county-level socioeconomic status measures, Mortality Disparities in American Community study

	Census tract-level characteristic	County-level characteristic
**Individual-level characteristic**	OR	OR
Household income		
Above U.S. median	2.284	1.325
Poverty level		
≤ 100% FPL	2.176	1.209
Education		
Has high school degree	2.370	1.214
Has college degree	2.513	1.364
Employment/occupation		
Is unemployed^a^	1.616	1.137
Employed in blue-collar industry^b^	1.324	1.030
Other characteristics		
Owns home	2.663	1.200
Foreign born	3.159	1.988

For household income, education, and owns home, having a value of 1 was considered low risk for health outcomes, while having a value of 0 was considered high risk; in contrast, for poverty, employment/occupation, and foreign born, having a value of 1 was considered high risk, while having a value of 0 was considered low risk. Each row is a different logistic regression model. Models included a control variable for county population. All *p* < .0001

*OR* odds ratio, *FPL* federal poverty level

^a^Unemployment was measured among participants who reported being in the workforce (e.g., excluding retirees)

^b^Employment in a blue-collar industry was measured among participants who reported being employed (i.e., excluding unemployed participants)

### Effects of misclassification of individual- and area-level SES characteristic on estimates of SES-mortality association

Mortality was associated with all SES characteristics across all socioecological levels (Table 5; all *p* < .0001). Across all indicators, the association was stronger for individual-level measures compared to the census tract- or county-level measures. For example, participants with a household income above the US median were less likely to die of any cause over the 7-year follow-up period than were other participants (OR = 0.318, 95% confidence interval [CI] = 0.315–0.321). This protective relationship was also seen but attenuated when comparing people living in high-income versus low-income census tracts (OR = 0.782, 95% CI = 0.776–0.789) or counties (OR = 0.803, 95% CI = 0.796–0.810).

**Table 5:** Associations between individual-, census tract-, and county-level indicators of socioeconomic status with all-cause mortality over 7-year follow-up, Mortality Disparities in American Communities study

	Individual-level characteristic		Census tract-level characteristic		County-level characteristic	
	OR	95% CI	OR	95% CI	OR	95% CI
Household income						
Above US median	0.318	(0.315–0.321)	0.782	(0.776–0.789)	0.803	(0.796–0.810)
Poverty level						
≤ 100% FPL	1.271	(1.255–1.287)	1.005	(1.004–1.005)	1.013	(1.012–1.013)
Education						
Has high school degree	0.395	(0.391–0.399)	0.838	(0.831–0.844)	0.895	(0.887–0.902)
Has college degree	0.480	(0.475–0.486)	0.994	(0.993–0.994)	0.989	(0.989–0.989)
Employment/occupation						
Is unemployed^a^	0.356	(0.344–0.368)	1.092	(1.084–1.101)	1.112	(1.103–1.121)
Employed in blue-collar industry^b^	0.284	(0.282–0.286)	1.006	(1.006–1.006)	1.015	(1.014–1.015)
Other characteristics						
Owns home	0.799	(0.791–0.806)	0.999	(0.999–1.000)	1.002	(1.002–1.003)
Foreign born	0.536	(0.527–0.544)	0.867	(0.859–0.875)	0.859	(0.852–0.867)

For household income, education, and owns home, having a value of 1 was considered low risk for health outcomes, while having a value of 0 was considered high risk; in contrast, for poverty, employment/occupation, and foreign born, having a value of 1 was considered high risk, while having a value of 0 was considered low risk. Each cell is a different logistic regression model. Models included a control variable for county population. All *p* < .0001

*OR* odds ratio, *CI* confidence interval, *FPL* federal poverty level

^a^Unemployment was measured among participants who reported being in the workforce (e.g., excluding retirees)

^b^Employment in a blue-collar industry was measured among participants who reported being employed (i.e., excluding unemployed participants)

Generally, the direction of the associations between the SES characteristics was consistent for individual- and area-level measures. However, unemployment and blue-collar occupation were two notable exceptions. At the individual level, participants who were unemployed or worked in a blue-collar industry were less likely than others to die over the follow-up period, but at the area level, people who were living in census tracts or counties with higher levels of unemployment or higher proportions of people working in blue-collar industries were more likely to die than people living in other areas.

## Discussion

In this analysis of ~ 3.5 million people, we found moderate agreement of SES across individual, census tract, and county socioecological levels. High area-level SES was associated with higher individual-level SES. However, some misclassification of individual-level SES using area-level measures was noted, and likely contributed to progressively attenuated estimates of the SES-mortality associations at larger socioecological levels.

There was variation in performance when comparing individual to area measures [36]. The census tract-level SES characteristics more closely approximated individual-level measures than did county-level characteristics. Note that these categories reflected high- or low-risk categories based on SES. Comparing the census tract and county variables to the “gold standard” of individual SES, we found that the two area levels performed similarly to each other. For example, the associations between SES and mortality were similar for census tract- and county-level variables (e.g., for household income, ORs were 0.782 and 0.803, respectively). Despite these similarities, other research studies suggest that SES measures observed at smaller geographic units are generally more precise proxies for individual SES [23, 25, 37, 38]. Studies aiming to approximate individual-level SES may be more successful using finer-grained area measures [30]; however, the increased precision of census tracts versus county measures did not translate into large differences in the present study.

Household income and foreign born had some of the largest ORs for concordance across levels (indicating greater agreement for individual- compared to area-level measures), while employment/occupation indicators (i.e., unemployment and occupation in a blue-collar industry) performed worse. Perhaps this reflects the fact that area-level unemployment rates are low (0% to < 20%), but experiencing individual-level unemployment has profound social and economic consequences, making their implications quite different. This finding provides additional guidance for researchers: Using indicators such as household income (perhaps the most common SES indicator) or percent foreign born will more precisely approximate individual-level values than employment/occupation indicators. In addition, we also found that individual- and area-level associations with mortality varied in direction for employment/occupation (Table 5). One previous study [39] examining the simultaneous associations between mortality and individual-/area-level unemployment concluded that area-level unemployment is confounded with other mechanisms that affect health (e.g., pollution), illustrating the use of area-level indicators as contextual factors rather than proxies for individual-level SES. Thus, extreme caution is warranted in evaluating studies using employment/occupation indicators to approximate individual-level SES.

Overall, area-level SES indicators had limitations as proxies for individual-level SES [18]. Studies using area-level indicators of SES may systematically underestimate the (individual) SES-mortality association due to misclassification between individual- and area-level measures. Nonetheless, area-level measures of SES are important in their own regard, since they can reflect other health-related characteristics, such as conditions in the social and physical environment [16, 40, 41]. Additional studies are needed to enhance understanding of how area-level SES status operates independently of individual attributes with respect to health outcomes.

Study strengths include the use of a high-quality dataset with exceptionally large sample size and national geographic scope. Millions of participants were successfully geocoded to census tracts and/or counties, allowing us to examine SES measures across multiple socioecological levels. We analyzed some of the most commonly used indicators of SES related to income, education, and occupation. A limitation was that, for data stability, we used 5-year averages for the area-level SES measures; 1-year indicators may be more relevant for comparisons to individual-level SES. Self-reported measures of SES may also be biased [42]. The individual- and area-level measures of SES used in the current study were based on self-report, so any bias would likely affect data across socioecological level similarly, limiting differential impact. In addition, we only analyzed binary measures of SES, although studies of SES and health often use alternative categorizations (e.g., quartiles, quintiles). In our data, some variables were only available as binary indicators (e.g., foreign born); for simplicity, we arranged all the variables in a binary structure. Given that some SES indicators are more commonly used as continuous or ordinal variables, additional research is needed to understand the degree of concordance of these measures across multiple levels of aggregation, as well as the potential impact on estimates of the associations with mortality. As noted above, correlations between individual- and area-level indicators were restricted statistically by the process of aggregation; however, these correlations provide insight into defining people as high or low risk based on SES.

## Conclusions

Studies examining associations between SES and health face many challenges, most importantly, the frequent unavailability of individual-level data on SES. While using area-level measures of SES as proxies for individual-level SES may be an attractive solution, the current study shows that the validity of these indicators is suboptimal. Considerable misclassification was seen among individual-, census tract-, and county-level indicators of SES. The magnitude of association with mortality was attenuated with area-level data, and in the case of employment/occupation, the direction of associations was opposite that for individual-level data. For studies that cannot measure individual-level SES, the best options may be use of area-level SES (1) measured at smaller geographic units and (2) based on indicators that perform well across socioecological levels, e.g., household income. Importantly, area-level SES provides useful contextual information relevant to health beyond individual-level SES. Additional research is needed to understand the validity of area-level SES proxies across demographic subgroups and health outcomes, and to parse the role of individual- versus area-level SES in public health.

## Data Availability

Several options are available for accessing the Mortality Disparities in American Communities data (https://www.census.gov/topics/research/mdac.Data_Availability.html). These options include indirect access (working with statisticians from the US Census Bureau) and direct access (working through a Federal Statistical Research Data Center or at the US Census Bureau, or through a limited data file).

## Abbreviations

ACS: American Community Survey
CI: Confidence interval
MDAC: Mortality Disparities in American Communities
NDI: National Death Index
OR: Odds ratio
SES: Socioeconomic status

## References

[1] Oakes JM, Rossi PH (2003). The measurement of SES in health research: current practice and steps toward a new approach. Soc Sci Med.

[2] Powers MG (1982). Measures of socioeconomic status: an introduction. Measures Socioeconomic Status.

[3] Krieger N, Williams DR, Moss NE (1997). Measuring social class in US public health research: concepts, methodologies, and guidelines. Annual Review of Public Health..

[4] Buell P, Dunn JE, Breslow L (1960). The occupational-social class risks of cancer mortality in men. J Chronic Dis.

[5] Freeman HP (1989). Cancer in the socioeconomically disadvantaged. CA: A Cancer Journal for Clinicians..

[6] Adler NE, Boyce WT, Chesney MA, Folkman S, Syme SL (1993). Socioeconomic inequalities in health: no easy solution. Jama..

[7] Subramanian SV, Kawachi I (2004). Income inequality and health: what have we learned so far?. Epidemiol Rev.

[8] Boscoe FP, Henry KA, Sherman RL, Johnson CJ (2016). The relationship between cancer incidence, stage and poverty in the United States. International Journal of Cancer..

[9] Moore JC, Welniak EJ (2000). Income measurement error in surveys: a review. J Official Stat.

[10] Chen JT, Kaddour A, Krieger N (2008). Implications of missing income data. Public Health Rep.

[11] Krieger N (1992). Overcoming the absence of socioeconomic data in medical records: validation and application of a census-based methodology. American Journal of Public Health..

[12] US Census Bureau (2018). American Community Survey (ACS).

[13] Kish JK, Yu M, Percy-Laurry A, Altekruse SF (2014). Racial and ethnic disparities in cancer survival by neighborhood socioeconomic status in Surveillance, Epidemiology, and End Results (SEER) Registries. Journal of the National Cancer InstituteMonographs..

[14] Cubbin C, Hadden WC, Winkleby MA (2001). Neighborhood context and cardiovascular disease risk factors: the contribution of material deprivation. Ethnic Dis.

[15] Diez Roux AV (2001). Investigating neighborhood and area effects on health. American Journal of Public Health..

[16] Ellen IG, Mijanovich T, Dillman KN (2001). Neighborhood effects on health: exploring the links and assessing the evidence. J Urban Affair.

[17] Hanley GE, Morgan S (2008). On the validity of area-based income measures to proxy household income. BMC Health Serv Res.

[18] Geronimus AT, Bound J, Neidert LJ (1996). On the validity of using census geocode characteristics to proxy individual socioeconomic characteristics. Journal of the American Statistical Association..

[19] Gomes FS, Vasconcellos MT, Anjos LA (2009). The use of income information of census enumeration area as a proxy for the household income in a household survey. Population Health Metrics.

[20] Conroy SM, Shariff-Marco S, Koo J, Yang J, Keegan TH, Sangaramoorthy M (2017). Racial/ethnic differences in the impact of neighborhood social and built environment on breast cancer risk: the Neighborhoods and Breast Cancer Study. Cancer Epidemiol Biomarker Prev.

[21] Kaiser P, Auchincloss AH, Moore K, Sanchez BN, Berrocal V, Allen N (2016). Associations of neighborhood socioeconomic and racial/ethnic characteristics with changes in survey-based neighborhood quality, 2000-2011. Health & Place..

[22] Hastert TA, Beresford SA, Sheppard L, White E (2015). Disparities in cancer incidence and mortality by area-level socioeconomic status: a multilevel analysis. J Epidemiol Commun Health.

[23] Diez-Roux AV, Kiefe CI, Jacobs DR, Haan M, Jackson SA, Nieto FJ (2001). Area characteristics and individual-level socioeconomic position indicators in three population-based epidemiologic studies. Ann Epidemiol.

[24] Thomas AJ, Eberly LE, Davey Smith G, Neaton JD (2006). Multiple risk factor intervention trial research G. ZIP-code-based versus tract-based income measures as long-term risk-adjusted mortality predictors. Am J Epidemiol.

[25] Lovasi GS, Moudon AV, Smith NL, Lumley T, Larson EB, Sohn DW (2008). Evaluating options for measurement of neighborhood socioeconomic context: evidence from a myocardial infarction case-control study. Health & place..

[26] Yin D, Morris C, Allen M, Cress R, Bates J, Liu L (2010). Does socioeconomic disparity in cancer incidence vary across racial/ethnic groups?. Cancer Causes & Control : CCC..

[27] Wan N, Zhan FB, Cai Z (2011). Socioeconomic disparities in prostate cancer mortality and the impact of geographic scale. Southern Medical Journal..

[28] Roblin DW (2013). Validation of a neighborhood SES index in a managed care organization. Medical Care..

[29] Pardo-Crespo MR, Narla NP, Williams AR, Beebe TJ, Sloan J, Yawn BP (2013). Comparison of individual-level versus area-level socioeconomic measures in assessing health outcomes of children in Olmsted County, Minnesota. Journal of Epidemiology and Community Health..

[30] Krieger N, Chen JT, Waterman PD, Soobader MJ, Subramanian SV, Carson R (2002). Geocoding and monitoring of US socioeconomic inequalities in mortality and cancer incidence: does the choice of area-based measure and geographic level matter?: the Public Health Disparities Geocoding Project. American Journal of Epidemiology..

[31] Galobardes B, Shaw M, Lawlor DA, Smith GD, Lynch J, Oakes JM, Kaufman JS (2006). Indicators of socioeconomic position. Methods in social epidemiology.

[32] Bureau USC (2017). How the Census Bureau measures poverty.

[33] Bureau USC (2003). Occupations: 2000.

[34] Robinson WS (2009). Ecological correlations and the behavior of individuals. International Journal of Epidemiology..

[35] Piantadosi S, Byar DP, Green SB (1988). The ecological fallacy. American Journal of Epidemiology..

[36] Braveman PA, Cubbin C, Egerter S, Chideya S, Marchi KS, Metzler M (2005). Socioeconomic status in health research: one size does not fit all. Jama..

[37] Nkosi TM, Parent ME, Siemiatycki J, Pintos J, Rousseau MC (2011). Comparison of indicators of material circumstances in the context of an epidemiological study. BMC Med Res Methodol.

[38] Southern DA, Ghali WA, Faris PD, Norris CM, Galbraith PD, Graham MM (2002). Misclassification of income quintiles derived from area-based measures. A comparison of enumeration area and forward sortation area. Can J Public Health.

[39] Tapia Granados JA, House JS, Ionides EL, Burgard S, Schoeni RS (2014). Individual joblessness, contextual unemployment, and mortality risk. Am J Epidemiol.

[40] Macintyre S, Ellaway A, Cummins S (2002). Place effects on health: how can we conceptualise, operationalise and measure them?. Soc Sci Med.

[41] Anderson RT, Sorlie P, Backlund E, Johnson N, Kaplan GA (1997). Mortality effects of community socioeconomic status. Epidemiology.

[42] Delgado-Rodriguez M, Llorca J (2004). Bias. Journal of Epidemiology and Community Health..

